# Effects of Belt Electrode-Skeletal Muscle Electrical Stimulation on Lower-Extremity Muscle Temperature

**DOI:** 10.7759/cureus.81105

**Published:** 2025-03-24

**Authors:** Katsuyoshi Tanaka, Shota Oda, Michio Wachi

**Affiliations:** 1 Department of Physical Therapy, Bukkyo University, Kyoto, JPN; 2 Department of Rehabilitation, Kochi Medical School Hospital, Kochi, JPN

**Keywords:** belt electrode-skeletal muscle electrical stimulation, electrical stimulation, high frequency, low frequency, muscle temperature, passive warm-up

## Abstract

Introduction

Warm-ups are essential for optimizing physiological readiness before physical activity, and active warm-up methods increase muscle temperature and neuromuscular activation. However, excessive exertion can lead to fatigue. Belt electrode-skeletal muscle electrical stimulation (B-SES) enhances muscle flexibility and prevents atrophy; however, its effectiveness in increasing muscle temperature as a passive warm-up strategy remains unclear. Therefore, we aimed to examine the effects of B-SES on lower limb muscle temperature.

Methods

This cross-sectional study comprised 20 healthy adults randomly assigned to either a low-frequency (LF; 4 Hz) or high-frequency (HF; 20 Hz) B-SES condition. Electrical stimulation was applied to the dominant lower extremity for 10 min, and the muscle temperature was measured every minute during and after stimulation. Temperature changes over time and between groups were analyzed using repeated-measures two-way analysis of variance (ANOVA). Repeated-measures one-way ANOVAs were conducted as post hoc analyses.

Results

Muscle temperature significantly increased over time in LF and HF conditions (p < 0.01), starting from 2 min of stimulation (at 2 min, LF: 0.24 ± 0.04℃; HF: 0.18 ± 0.04℃). However, no significant differences were observed between the two stimulation conditions (p = 0.09). The median fatigue rating on the visual analog scale was 31.5 mm (interquartile range, 14.8-60.8 mm).

Conclusion

B-SES effectively increased the muscle temperature within a short duration, suggesting its potential as an efficient passive warm-up method. Further research is required to explore its functional benefits in athletic performance and rehabilitation settings.

## Introduction

Warm-up is a fundamental preparatory phase preceding physical activity. It is designed to optimize physiological readiness for exercise. Active warm-up, characterized by voluntary muscle contractions, elevates muscle temperature and activates the nervous system, thereby enhancing performance [1-5]. It includes intense contractions that can enhance post-activation potentiation, which may increase muscle fiber water content and muscle activation [6]. An increase in muscle temperature is essential for effective warm-up, as it enhances enzymatic activity, improves nerve condition velocity, and reduces muscle stiffness, all of which contribute to optimized muscle function and reduced injury risk [7,8]. A previous study showed that even a small increase in skeletal muscle temperature can rapidly activate contractile proteins and improve muscle performance [9]. However, excessive engagement in active warm-up may induce fatigue and increase the risk of tissue damage, ultimately compromising performance.

In contrast, passive warm-up methods, such as heat therapy, offer a viable solution to mitigate these issues by increasing muscle temperature without inducing fatigue or excessive strain. Previous studies have investigated the effects of heat therapy on muscle temperature and performance. Petrofsky et al. [10] examined the impact of different heating modalities on deep-tissue temperature and highlighted the critical role of body fat and blood flow in heat transfer. However, findings regarding the efficacy of heat therapy in enhancing performance remain inconsistent.

Electrical stimulation has recently been explored as an alternative warm-up method because it can activate muscles without requiring voluntary effort. Notably, some studies suggest that electrical stimulation can enhance blood flow and neuromuscular activation, potentially leading to benefits similar to those of active warm-up [11,12]. Of these electrical stimulation methods, belt electrode skeletal muscle electrical stimulation (B-SES) is an efficient method for electrical stimulation of the entire lower extremities [13]. This approach enhances muscle flexibility and prevents atrophy [14,15], suggesting its potential as a passive warm-up technique. However, evidence of its effectiveness as a passive warm-up strategy, particularly in terms of its impact on muscle temperature, remains unknown. Thus, examining the effects of B-SES on muscle temperature before evaluating its effectiveness as a passive warm-up strategy is crucial.

Therefore, this study aimed to investigate the effects of B-SES on muscle temperature, providing fundamental evidence for its potential use as an effective passive warm-up method. Understanding its impact on muscle temperature may contribute to optimizing warm-up protocols, reducing fatigue-related risks, and improving athletic performance and rehabilitation strategies.

## Materials and methods

Participants

Considering potential attrition, we recruited 20 healthy adults from a university in Japan between December 2024 and February 2025. Participants were eligible if they were ≥ 18 years old. The exclusion criteria were: (1) currently undergoing medical treatment for any illness or injury, and (2) a history of stroke, cardiovascular disease, or psychiatric disorders.

This study employed a cross-sectional design in which data were collected from each participant on a single occasion.

Based on *a priori *power analysis using G*Power 3.1 (Heinrich Heine University, Dusseldorf, Germany) [16], a minimum sample size of 14 participants was required for repeated-measures analysis of variance (ANOVA). The calculation was based on a partial eta squared (η2) effect size of 0.06 (medium effect), an alpha level of 0.05, and a power (1−β) of 0.80. The study design included a between-subjects factor with two levels and a within-subjects factor with 11 repeated measures. The assumed correlation among repeated measures was set at 0.5, and sphericity was assumed. This approach followed the established recommendations for power analysis in repeated-measures designs [17,18]. Participants were randomly assigned to either the low-frequency (LF) or high-frequency (HF) groups. The Human Research Ethics Review Committee of Bukkyo University approved this study (2024-31-B). This study was conducted in accordance with the principles of the Declaration of Helsinki, and informed consent was obtained from all participants before the study. This clinical study was registered with the University Hospital Medical Information Network in Japan (UMIN000057180).

Procedures

Participants were interviewed to obtain demographic data (age, sex, height, and weight). Before electrical stimulation, the temperature of the dominant calf muscle at baseline was measured in all participants using Core temp CTM-205 (Terumo Co., Ltd., Tokyo, Japan), a non-invasive device that continuously measures muscle temperature beneath the probe. The probe was attached to the middle of the gastrocnemius medialis muscle belly on the dominant side following a previous study [19]. The medial gastrocnemius is a common site of muscle and fascial injuries in sports settings [20,21], making it clinically relevant for evaluating the effects of warm-up. In addition, it has relatively less subcutaneous fat, allowing for stable and reproducible temperature measurements. The stimulation type was randomly selected as either LF or HF and applied to the dominant lower extremities for 10 min. Muscle temperature was measured every minute during and after stimulation. After stimulation, the participants reported their level of fatigue using a visual analog scale.

Stimulation

**Figure 1:**
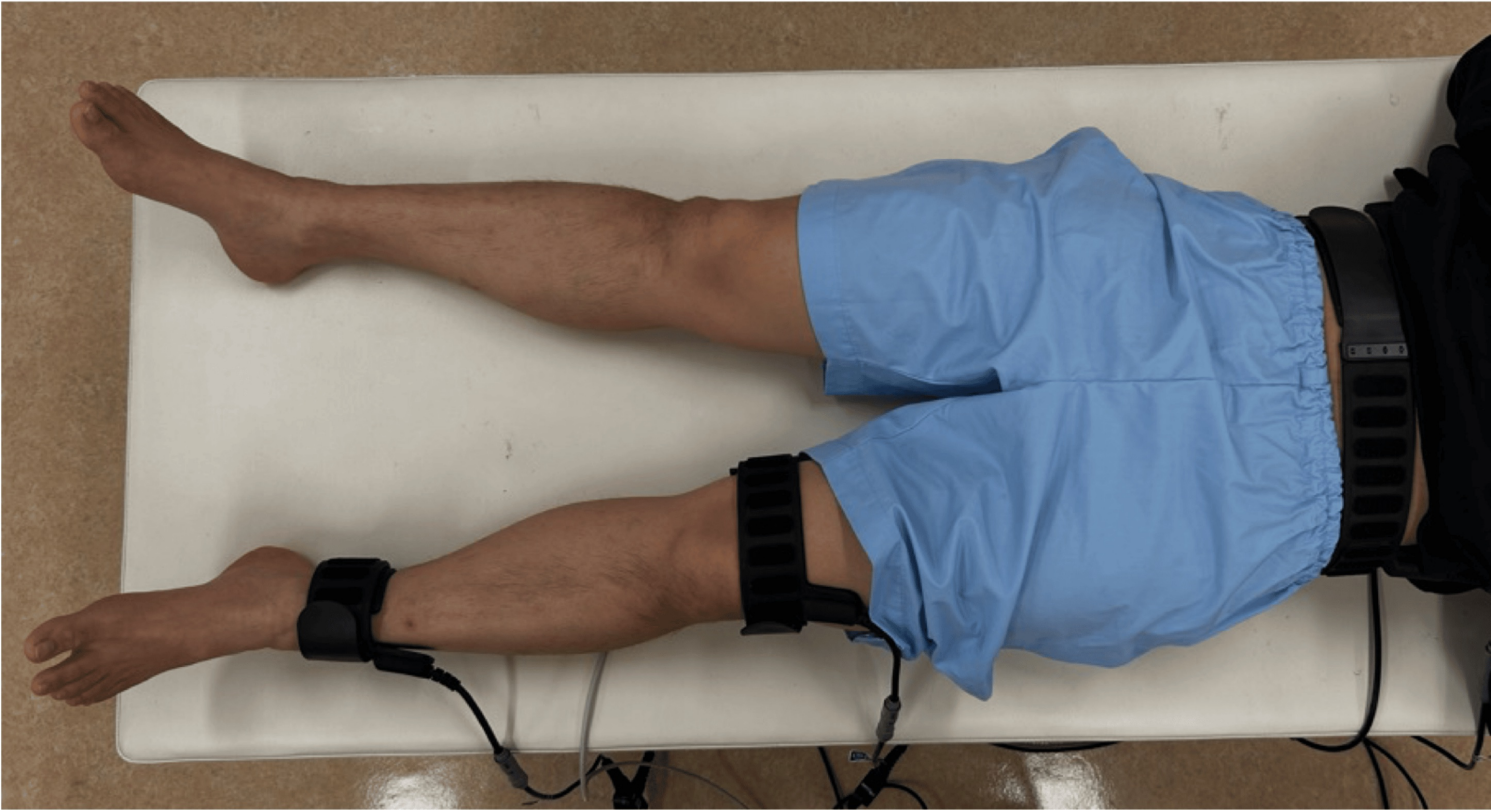
Belt-electrode skeletal muscle electrical stimulation (B-SES). The B-SES was applied to dominant lower extremity.

B-SES (G-TES®; Homer Ion, Tokyo, Japan) was applied in both groups. Belt electrodes were attached at three locations: around the participant’s waist, above the knee, and above the ankle on the dominant side (Figure 1).

Electrical muscle stimulation was applied to all lower extremities between the belts. The two stimulation conditions were as follows: (1) LF group (stimulation frequency, 4 Hz; pattern: repeated twitching) and (2) HF group (stimulation frequency, 20 Hz; pattern: 5 s of strong contractions followed by 2 s of rest). The electrical stimulation intensity was adjusted to the highest level that produced visible muscle contraction while remaining within the participant’s tolerance level.

Statistical analyses

Appropriate statistical tests were selected based on data distribution and variable type to compare participant characteristics between the two groups. The tests include the Mann-Whitney U test for non-normally distributed continuous variables, the independent t-test for normally distributed continuous variables, and Fisher’s exact test for categorical variables. Levene’s test was conducted to assess the homogeneity of variances before applying parametric tests. If heteroscedasticity was detected, Welch’s-test was applied instead of the independent t-test. Changes in muscle temperature over time between the LF and HF groups were analyzed using repeated-measures two-way ANOVA. In addition, post hoc analyses were performed for each condition to assess time-dependent changes using repeated-measures one-way ANOVA. Dunnett’s test was used for multiple comparisons with baseline values serving as controls. All statistical analyses were conducted using the Statistical Package for Social Sciences version 26.0 (IBM Corp., Armonk, U.S.A.), with the significance level set at 0.05.

## Results

Table 1 shows participants’ characteristics. No significant differences were observed between the LF and HF groups. There were no significant differences in the mean stimulation intensities between the LF and HF groups for both the thigh (LF: 2.3 ± 0.8 mA, HF: 3.3 ± 1.4 mA, p = 0.11) and the lower leg (LF: 1.0 ± 0.5 mA, HF: 0.9 ± 0.3 mA, p = 0.83). The median of fatigue was 31.5 mm (Q1-Q3, 14.8-60.8 mm).

**Table 1:** Characteristics of the participants Note: Data are presented as mean ± SE for normally distributed continuous variables, median (Q1-Q3) for non-normally distributed continuous variables, and n (%) for categorical variables. ^a^Assessed by Mann-Whitney U test; ^b^Assessed by independent t-test; ^c^Assessed by Fisher’s exact test LF: low-frequency group (4 Hz); HF: high-frequency group (20 Hz)

	All (n = 20)	LF group (n = 10)	HF group (n = 10)	Test static	p-value
Age (years)	21.0 (20.0-22.0)	21.0 (20.3-22.0)	21.5 (20.0-22.0)	47.00	0.85^a^
Female (n, %)	9 (45%)	3 (30%)	6 (60%)	1.82	0.19^c^
Height (cm)	164.6 ± 2.0	167.2 ± 2.3	161.9 ± 3.1	−1.37	0.14^b^
Weight (kg)	54.5 (52.8-62.0)	55.5 (54.0-62.8)	54.5 (50.0-58.0)	63.00	0.35^a^

No significant difference was observed in muscle temperature at baseline between groups (LF, 34.7 ± 0.7℃; HF, 34.5 ± 1.1℃). A repeated two-way ANOVA revealed a significant main effect of time (F = 28.01, p < 0.01, η2 = 0.97), indicating that muscle temperature changed significantly over time. However, the main effect of the groups was not significant (F = 3.20, p = 0.09, η2 = 0.15), suggesting that the two conditions of stimulation did not differentially influence muscle temperature. In addition, the interaction effect between time and groups was not significant (F = 1.36, p = 0.33, η2 = 0.60).

**Figure 2:**
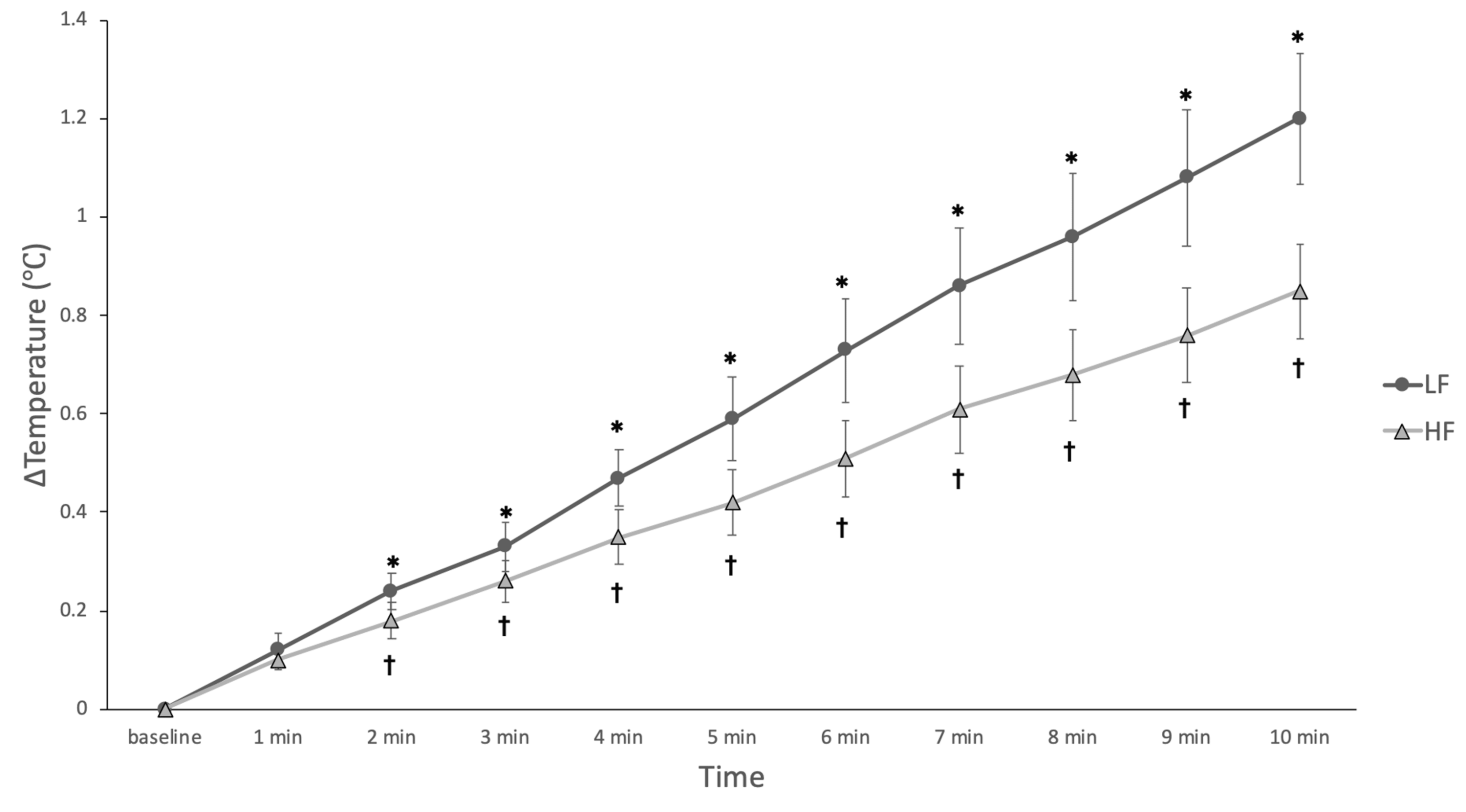
Increasing of muscle temperature during each electrical stimulation. Time-dependent changes were assessed using repeated-measures ANOVA, followed by Dunnett’s test for multiple comparisons. ^*^Significant differences compared to baseline in LF group; ^†^Significant differences compared to baseline in HF group. LF: low-frequency group (4 Hz); HF: high-frequency group (20 Hz)

Figure 2 shows the increase in muscle temperature during stimulations. Post hoc comparisons using Dunnett’s test revealed that for both conditions of electrical stimulation, muscle temperature showed a significant increase compared to baseline from 2 min onward. At 2 minutes, the temperature increase was statistically significant for the LF (mean = 0.24℃, SE = 0.04℃, 95% confidence interval (CI) = 0.01-0.47℃, p = 0.04) and HF groups (mean = 0.18℃, SE = 0.04℃, 95% CI = 0.04-0.32℃, p < 0.01).

## Discussion

In this study, we investigated the effects of B-SES on lower-extremity muscle temperature using LF and HF stimulation. Our findings showed that LF and HF conditions significantly increased muscle temperature, beginning at 2 min of stimulation, with no significant differences between the two stimulation conditions.

Our findings suggest that B-SES effectively increases muscle temperature within a short duration, and this temperature increase may have implications for neuromuscular function. However, our study did not directly assess neuromuscular activation or performance outcomes, so further research is needed to evaluate these effects. Meanwhile, previous studies have suggested that elevated muscle temperature is associated with increased muscle contraction velocity and power output, which are critical components of warm-up strategies [1,3,9,22]. These improvements are thought to result from an increase in nerve conduction velocity associated with elevated muscle temperature, which enhances the efficacy of cross-bridge cycling within muscle fibers [23,24].

Furthermore, elevated muscle temperature has been linked to a reduction in muscle stiffness and an increase in muscle compliance, which may contribute to improved force transmission and movement efficiency [11,25]. The increase in muscle temperature observed in this study was lower than that reported in previous studies. However, B-SES may facilitate neuromuscular activation due to the effects of electrical muscle stimulation [12,26,27]. Thus, while our findings demonstrate that B-SES effectively increases muscle temperature, its direct impact on neuromuscular function remains to be investigated in future studies.

In addition, this study’s findings highlighted the potential of B-SES as a passive warm-up method. Traditional active warm-up strategies can elevate muscle temperature but may also induce fatigue and increase the risk of tissue damage. A previous study has shown that low-intensity neuromuscular electrical stimulation enhances muscle strength and cardiovascular fitness [15], supporting the feasibility of B-SES as a passive warm-up tool. Furthermore, the fatigue induced by B-SES in this study was lower than that previously reported [23], suggesting that B-SES may serve as an effective alternative to the traditional warm-up strategy for neuromuscular activation without exacerbating fatigue.

This study had several limitations. First, the participant pool consisted solely of healthy young adults, and this may limit the generalizability of the findings to older individuals, athletes, or individuals with neuromuscular impairments. Additionally, the participant characteristics, such as sex distribution and body composition, were not sufficiently controlled. Given that subcutaneous fat thickness and muscle mass may influence electrical impedance and heat conduction, variations in these factors could have affected the muscle temperature response to B-SES.

Second, muscle temperature measurements were restricted to the gastrocnemius medialis, and it remains unclear whether similar effects occur in other muscles. However, given that electrical stimulation enhances circulation and increases temperature in multiple muscle groups [11,24,25], it is plausible that B-SES could elicit similar responses elsewhere.

Third, the measurement depth of the temperature probe was not validated against individual subcutaneous tissue thickness. Since subcutaneous fat may alter the effective depth of measurement, objective assessment, such as ultrasound imaging, should be used in future studies to confirm that the probe accurately reflects muscle tissue temperature.

Fourth, electrical stimulation was applied to only one lower extremity, and the effects on the non-stimulated extremity were not evaluated. Future studies should investigate the impact of B-SES on contralateral temperature changes and the effects of bilateral stimulation.

Finally, this study focused on temperature changes; however, the impact of B-SES on actual performance outcomes remains unexamined. Future research should assess whether the observed temperature elevations translate into meaningful improvements in athletic performance.

## Conclusions

This study demonstrated that B-SES increases lower extremity muscle temperature in a short duration, making it an efficient passive warm-up method. Further studies are needed to determine its broader applications and its influence on functional performance outcomes.
